# *Adamts18* deletion results in distinct developmental defects and provides a model for congenital disorders of lens, lung, and female reproductive tract development

**DOI:** 10.1242/bio.019711

**Published:** 2016-09-16

**Authors:** Dalya Ataca, Marian Caikovski, Alessandra Piersigilli, Alexandre Moulin, Charaf Benarafa, Sarah E. Earp, Yakir Guri, Corinne Kostic, Yvan Arsenivic, Raija Soininen, Suneel S. Apte, Cathrin Brisken

**Affiliations:** 1 Ecole Polytechnique Fédérale de Lausanne, ISREC, NCCR Molecular Oncology, Station 19, Lausanne CH-1015, Switzerland; 2 Jules-Gonin Eye Hospital, University of Lausanne, Avenue de France 15, Lausanne CH-1004, Switzerland; 3 Theodor Kocher Institute, University of Bern, Freiestrasse 1, Bern CH-3012, Switzerland; 4 Biomedical Engineering-ND20, Cleveland Clinic Lerner Research Institute, 9500 Euclid Ave., Cleveland, OH 44195, USA; 5 Biozentrum, University of Basel, Basel CH-4056, Switzerland; 6 Department of Pathology, Biocenter Oulu, University of Oulu, Oulu FIN-90014, Finland

**Keywords:** Adamts18, Metalloproteinase, Lens capsule, Lung development, Vaginal septum, Rete ovary

## Abstract

The ADAMTS family comprises 19 secreted metalloproteinases that cleave extracellular matrix components and have diverse functions in numerous disease and physiological contexts. A number of them remain ‘orphan’ proteases and among them is ADAMTS18, which has been implicated in developmental eye disorders, platelet function and various malignancies. To assess *in vivo* function of ADAMTS18, we generated a mouse strain with inactivated *Adamts18* alleles. In the C57Bl6/Ola background, *Adamts18*-deficient mice are born in a normal Mendelian ratio, and are viable but show a transient growth delay. Histological examination revealed a 100% penetrant eye defect resulting from leakage of lens material through the lens capsule occurring at embryonic day (E)13.5, when the lens grows rapidly. *Adamts18*-deficient lungs showed altered bronchiolar branching. Fifty percent of mutant females are infertile because of vaginal obstruction due to either a dorsoventral vaginal septum or imperforate vagina. The incidence of ovarian rete is increased in the mutant mouse strain. Thus, *Adamts18* is essential in the development of distinct tissues and the new mouse strain is likely to be useful for investigating ADAMTS18 function in human disease, particularly in the contexts of infertility and carcinogenesis.

## Abstract (precis)

**Summary:** We generated mice deficient for the secreted protease ADAMTS18 implicated in human disease and show that the gene is required in the development of the lens, lung, and the female reproductive tract.

## INTRODUCTION

ADAMTS18 is a member of the a disintegrin-like and metalloproteinase domain with thrombospondin type 1 motifs (ADAMTS) family of secreted Zn-dependent metalloproteinases (Kuno et al., 1997) that comprises 19 members, as reviewed in Apte (2009) and Kelwick et al. (2015). Like other metzincins, the catalytic activity of ADAMTS proteases depends on zinc ion binding in the active site, and unique to ADAMTS proteinases is a characteristically organized ancillary domain containing thrombospondin type 1 repeats (TSRs) (Shieh et al., 2008). ADAMTS proteases are synthesized as precursors with an N-terminal propeptide (Stanton et al., 2011), which is excised by proprotein convertases such as furin (Seidah et al., 2008). Some ADAMTS proteases process components of the extracellular matrix (ECM), such as fibrillar collagens, and others are implicated in turnover of the chondroitin sulphate proteoglycans: aggrecan and versican (Apte, 2009). ADAMTS13 specifically cleaves von Willebrand factor to maturity (Zander et al., 2015). Several family members, among them ADAMTS18, remain to be characterized with regard to their substrate profile.

Germ line mutations in *ADAMTS* genes are implicated in a number of human genetic disorders (Dubail and Apte, 2015); for example, mutations in *ADAMTS13* cause inherited thrombotic thrombocytopenic purpura, a condition associated with excessive platelet aggregation (Levy et al., 2001). Also, mutations in *ADAMTS10* or *ADAMTS17* underlie Weill–Marchesani syndrome and Weill–Marchesani-like syndrome, respectively, in humans, characterized by eye and skeletal abnormalities (Dagoneau et al., 2004; Morales et al., 2009). Mutations in *ADAMTS2*, which encodes a procollagen propeptidase, cause a subtype of Ehlers–Danlos syndrome, which is characterized by severe skin fragility (Colige et al., 2004); while ADAMTS5, 9, and 20 participate in limb morphogenesis, cardiovascular development, skin pigmentation and palatogenesis (McCulloch et al., 2009; Enomoto et al., 2010).

Single nucleotide polymorphisms in *ADAMTS18* were associated with reduced bone mineral density, which determines susceptibility to osteoporosis in three distinct ethnic groups (Xiong et al., 2009); and the C-terminus of ADAMTS18 was shown to induce platelet thrombus fragmentation (Li et al., 2009). In a patient suffering from early-onset severe retinal dystrophy, a homozygous missense mutation in one of the C-terminal thrombospondin repeats of ADAMTS18 was detected and implicated in photoreceptor function (Peluso et al., 2013).

To assess the *in vivo* function of *Adamts18*, we generated *Adamts18*-deficient mice and uncovered a requirement for ADAMTS18 in the development of eye, the lungs, and the female reproductive tract with implications for human disease.

## RESULTS

### Generation of *Adamts18* mutant mice

**Fig. 1.**
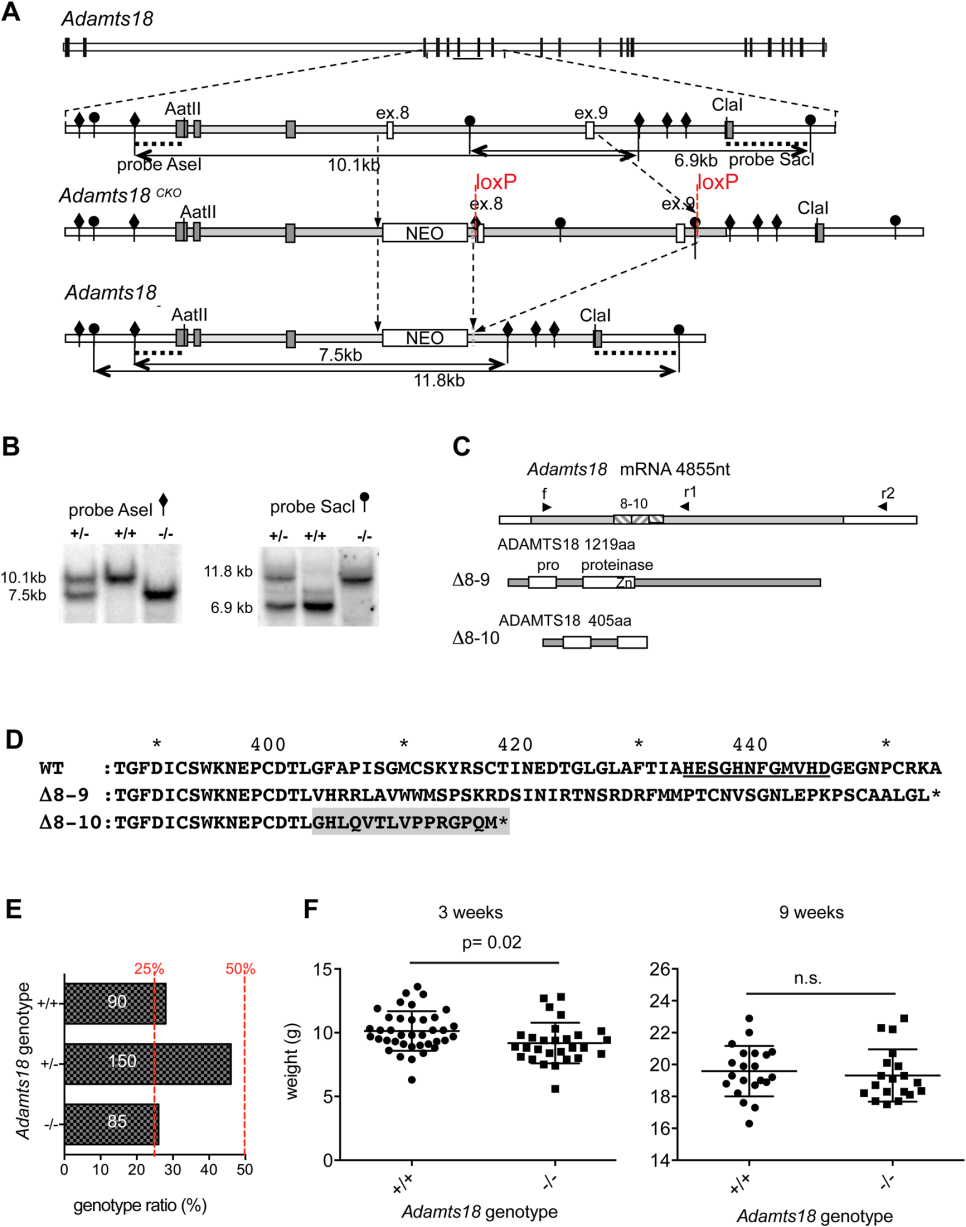
**Generation of *Adamts18*-deficient mice.** (A) Initially, a conditional knock out allele *Adamts18^CKO^* predicted to encode WT ADAMTS18 protein was created. The *ADAMTS18* region (gray) bordered by restriction sites *Aat* II and *Cla* I and containing exons 8 and 9 (blue) was replaced through homologous recombination by a modified sequence containing a neomycin resistance cassette (NEO) and two loxP sites flanking exons 8 and 9. The *Adamts18^KO^* allele was obtained by crossing male mice with the conditional *Adamts18^CKO^* allele to females of the *MMTV-Cre* line A (Wagner et al., 2001). Exons 8 and 9 (white), encoding for the Zn-binding site of the catalytic domain, were replaced by a neomycin cassette. In the blow-up, *Ase* I restriction sites are indicated by diamonds and *Sac* I restriction sites by lollipops. (B) The mutation changed the pattern of genomic DNA digestion with restriction endonucleases *Ase* I or *Sac* I revealed by Southern blotting of genomic DNA. (C) Schemes of predicted *Adamts18* transcripts (coding sequence in gray). The transcript can be amplified from both WT and *Adamts18*^−/−^ eyes using primer pairs f-r1 or f-r2 (see panel A). Sequencing of the mutant transcript revealed that in addition to the targeted exons 8 and 9 (striped) exon 10 (striped) is missing. (D) Translation of the expected *Adamts18*^KO^ (D8-9) and the cloned (D8-10) sequences indicates a frame shift resulting in a protein truncated at amino acid 405. (E) Frequency of *Adamts18* genotypes in offspring from 21 *Adamts18*^+/−^ mating pairs was determined. Columns represent the percentage of the registered genotypes; dashed red lines indicate expected frequency based on Mendelian inheritance. Numbers in the columns represent absolute numbers of pups for each genotype; WT: *n*=90, *Adamts18*^+/−^: *n*=150, *Adamts18*^−/−^: *n*=85. The significance of deviation from the expected Mendelian ratio was tested using chi-squared statistics as non-significant, *P*=0.32. (F) Weight of *Adamts18*^−/−^ females and their WT littermates at 3-weeks of age (*n*=38 and 28, respectively), *P*=0.018, two-tailed Student's *t*-test, and at 9-weeks of age (*n*=21 and 19), *P*=0.6, two-tailed Student's *t*-test.

To establish *Adamts18* function *in vivo*, we generated mice homozygous for an engineered allele lacking exons 8 and 9, which encode the conserved Zn-binding site of the predicted catalytic domain (Fig. 1A). To circumvent potential embryonic lethality, we initially generated a mouse strain with a conditional allele, *Adamts18*^CKO^. Germ line deletion was achieved by crossing the *Adamts18*^CKO^ males to females carrying an *MMTV-Cre* transgene, which is expressed in oocytes (Wagner et al., 2001). Southern blotting of tail DNA from offspring with two different probes confirmed successful deletion of exons 8 and 9, as well as parts of the flanking introns (Fig. 1B,C). The mutated *Adamts18* allele (*Adamts18^−^*) was transcribed with a shortened transcript, readily detected by RT-PCR in RNA extracted from eyes of homozygous mutant adult mice (*Adamts18*^−/−^) (Fig. S1A). Sequencing of the transcript revealed that, in addition to the targeted exons 8 and 9, the transcript also lacked exon 10, likely because the deletion of intronic sequence resulted in defective splicing. Translation of the nucleotide sequence of the predicted transcript lacking exons 8-9, or the cloned transcript lacking exons 8-10, indicated that either deletion caused a frame shift resulting in premature stop codons in the respective downstream sequence spliced to exon 7 (Fig. 1D,F). Thus, a putative ADAMTS18^−^ protein, were it to be translated, correctly folded, and secreted, retains maximally 405 of 1219 amino acids of the wild-type (WT) sequence comprising only the propeptide and the N-terminal half of the catalytic domain.

After backcrossing for 9 generations on the C57BL/6JOlaHsd background, approximately 28% of the offspring born to *Adamts18*^+/−^ intercrosses were *Adamts18*^−/−^ (of *n*=325), indicating no significant deviation from expected Mendelian ratios (*P*=0.32) (Fig. 1G). At birth, the homozygous mutant pups were indistinguishable from their WT and heterozygous littermates. At weaning, i.e. at 3 weeks of age, *Adamts18*^−/−^ pups were on average 93% of the weight of gender-matched littermates (Fig. 1H); at 9 weeks, weight was comparable between *Adamts18*^−/−^ and their WT littermates (Fig. 1I). Thus, *Adamts18* deletion causes a transient growth delay.

### The role of *Adamts18* in eye development

**Fig. 2.**
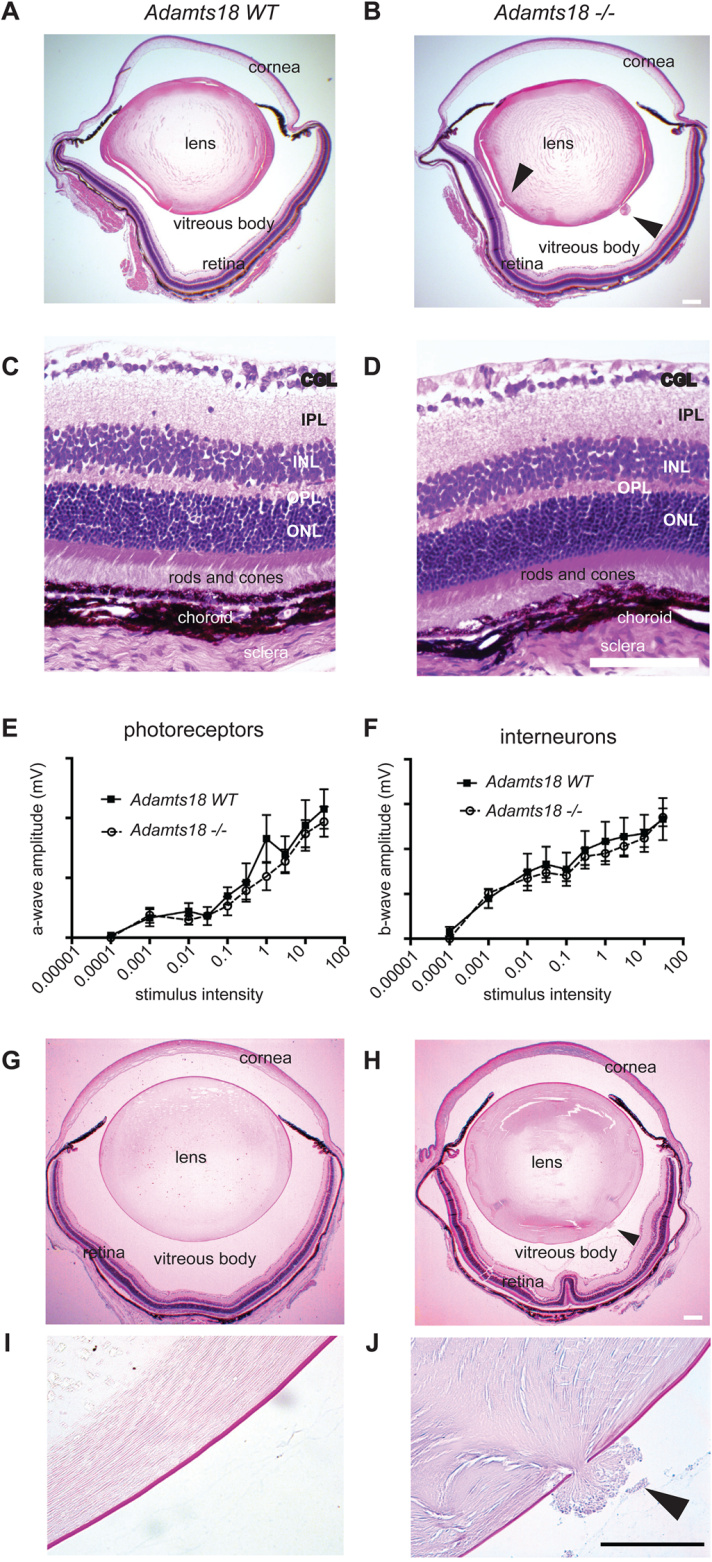
**Eye defect in *Adamts18*^−/−^ mice.** (A,B) Micrographs of H&E-stained histological sections of eyes of 8-week-old WT (A) and *Adamts18*^−/−^ (B) littermates. In the *Adamts18*^−/−^ eyes, there are extrusions of posterior lens material into the vitreous cavity (arrows) (B). Scale bar: 200 μm. (C,D) Higher magnification of retinas from WT (C) and *Adamts18*^−/−^ (D) littermates. GCL, ganglion cell layer; IPL, inner plexiform layer; INL, inner nuclear layer; OPL, outer plexiform layer (Donley and Thayer, 2013); ONL, outer nuclear layer. Scale bar: 200 μm. (E,F) Electroretinograms of WT (E) and *Adamts18*^−/−^ (F) littermates indicate comparable responses; *n*=4 each genotype (Xiong et al., 2009). (G,H) Micrographs of PAS-stained histological sections from eyes of 8-week-old WT (G) and *Adamts18*^−/−^ (H) littermates. Scale bar: 200 μm. (I,J) Higher magnification of the lens capsules of WT (I) and *Adamts18*^−/−^ (J) littermates. The arrowhead points to extruded lens material through a break in the posterior lens capsule (dark pink, PAS stain). Scale bar: 100 μm.

Because of the association of germ-line *ADAMTS18* mutations with eye disorders in humans (Chandra et al., 2014) and the observation that *Adamts18* mRNA expression is high in the mouse eye, in particular in the lens and retina (Fig. S1B), we dissected eyes from 2-month-old *Adamts18^−/−^* mice. Macroscopically, the eyes appeared normal. Hematoxylin and eosin (H&E) staining of histological sections revealed that the overall structure of the eyes was intact in *Adamts18^−/−^* mice (Fig. 2A,B). The retina was structurally normal (Fig. 2C,D) and its functional integrity as assessed by electroretinogram (ERG) was preserved (Fig. 2E,F). We observed breaks in the posterior lens capsule with extruded lens material in the mutants (Fig. 2B, arrowhead). The breaks in the lens capsule, that is rich in polysaccharides, are best highlighted with periodic acid-Schiff (PAS) staining (Fig. 2G,H,I,J). This phenotype was 100% penetrant (*n*=12), while in the WT eyes not a single extrusion was detected, and the *Adamts18^−/−^* eyes had 4-5 extrusions per eye.

**Fig. 3.**
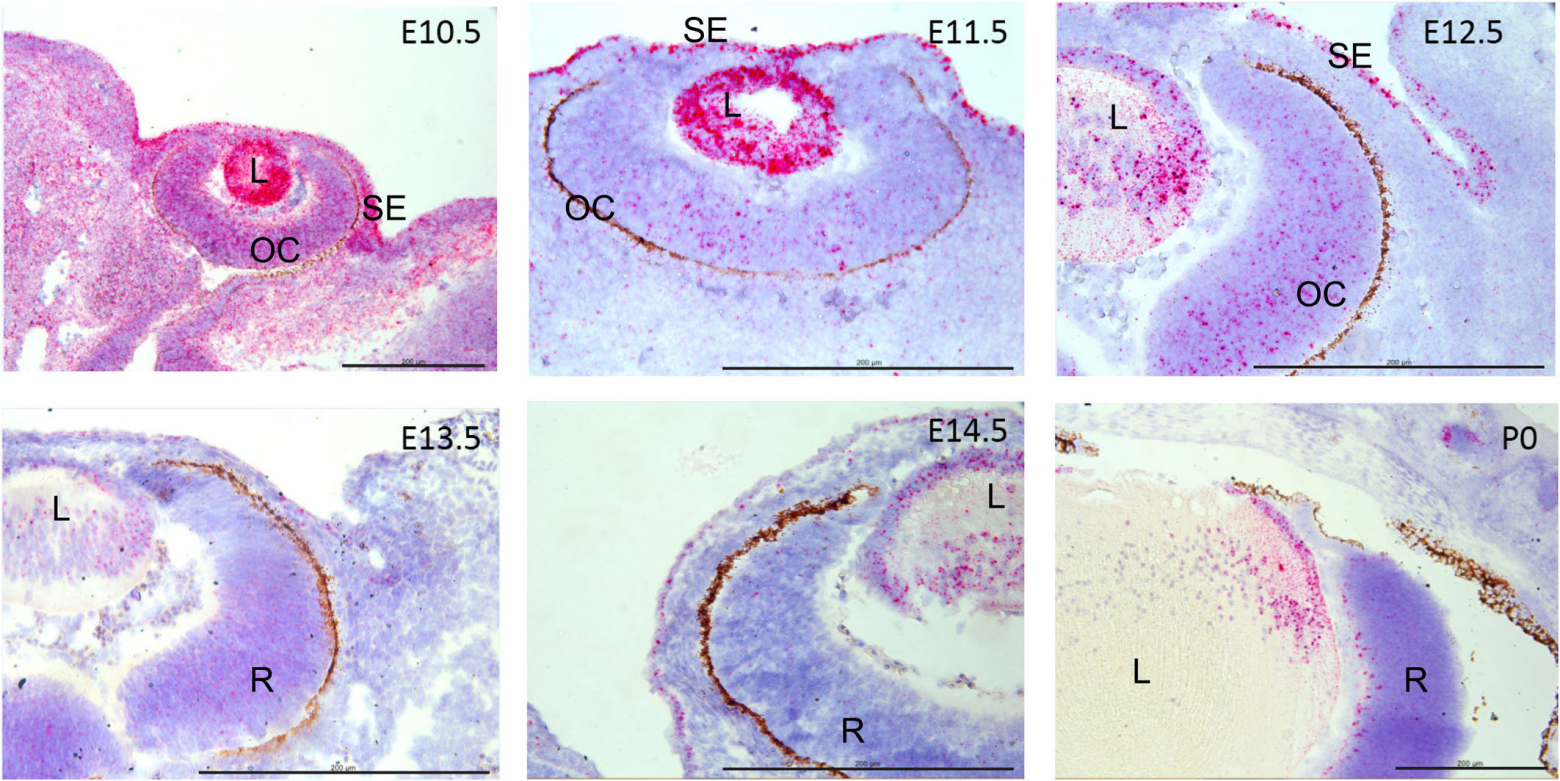
***Adamts18* mRNA localization during mouse eye development.** *Adamts18* mRNA, indicated by red dots overlying cells, is strongly expressed in the lens (L) throughout the development of the eye, with strongest expression at E10.5 and E11.5 and declining thereafter. At E10.5, E11.5, and E12.5, *Adamts18* mRNA is also expressed in the surface ectoderm (SE) and the optic cup (OC). At E13.5, E14.5, and at birth (P0), *Adamts18* mRNA is localized to lens nuclei and lens epithelium. At P0, *Adamts18* mRNA is expressed in the innermost layer of the developing retina (R), and the ganglion cell layer. Hematoxylin counterstain is blue. Scale bars: 200 µm.

**Fig. 4.**
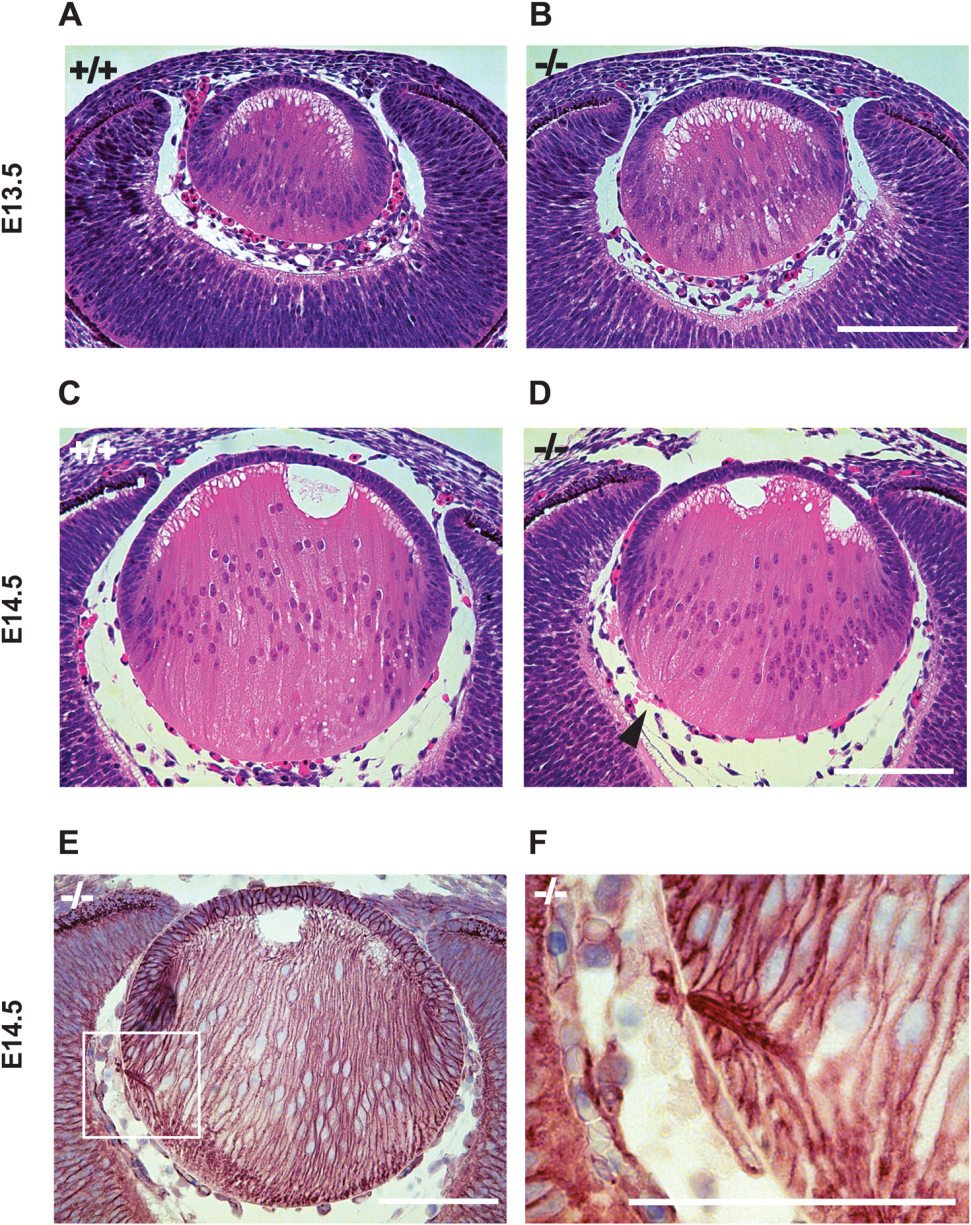
**Chronology of the *Adamts18*^−/−^ lens capsule defect.** H&E stained sections of eyes from WT (A,C) and *Adamts18*^−/−^ (B,D) embryos. At E13.5, the lens capsule is intact both in WT (*n*=9) and *Adamts18*^−/−^ (*n*=9) embryos (A,B). At E14.5 in the *Adamts18*^−/−^ embryos, protrusions of the lens substance (arrowhead) become evident (D). Note the rapid growth of the lens during the illustrated time period (A,B versus C,D). (E) Adjacent section stained with antibody against β-catenin, which reveals plasma membranes and highlights shape and orientation of fiber cells in *Adamts18*^−/−^ lens. (F) Higher magnification of the area indicated in (E). The extrusions are formed by projections of adjacent fiber cells (arrow) in *Adamts18*^−/−^ embryos. Scale bar: 100 μm.

Interestingly, among the different tissues in the eye, the lens showed the highest *Adamts18* transcript expression (Fig. S1B). To further investigate the spatial and temporal pattern of expression during mouse eye development, we localized *Adamts18* mRNA by *in situ* hybridization in eyes of WT embryos. *Adamts18* mRNA (indicated by red dots overlying cells) was strongly expressed in the lens throughout eye development, with strongest expression at E10.5 and E11.5. At E10.5, E11.5, and E12.5, *Adamts18* mRNA was also expressed throughout the eyelid, prospective cornea, and optic cup (Fig. 3). At E13.5, E14.5, and P0, *Adamts18* mRNA localized to the lens equator and anterior lens epithelial cells. At P0, *Adamts18* mRNA was also expressed in the inner layers of the developing retina. Because of its expression throughout embryonic eye development, we examined H&E-stained sections of eyes from embryos at different stages between the E11.5 and E14.5 period of lens development (Richard et al., 2001) in order to establish when the lens defects appeared. During this time period, the surface ectoderm, which is transformed into the lens epithelium, increases 12-fold in thickness (Danysh and Duncan, 2009). Until E13.5, both WT and *Adamts18^−/−^* littermates had intact lens capsules (*n*=10 for E13.5, *n*=6 for E11.5 and E12.5) (Fig. 4A,B). Between E13.5 and E14.5, when the developing eye grows rapidly and lens fiber cells proliferate rapidly (Kallifatidis et al., 2011), protruding cells became apparent in 100% of mutant eyes (*n*=10) (Fig. 4C,D). To visualize the cellular organization of the extrusions, we stained plasma membranes of fiber cells with an antibody against β-catenin, a protein associated with the cytosolic part of the plasma membranes. This revealed that each extrusion was formed by projections of plasma membranes of several adjacent lens fiber cells (Fig. 4F). Electron microscopy revealed that the WT and *Adamts18^−/−^* lens capsules were structurally undistinguishable, and showed that fiber cells, which were ordered in the WT, were entangled in the *Adamts18^−/−^* lens at the site of extrusions (Fig. S2A-D). This is different from a similar extrusion phenotype reported in perlecan mutant mice (Rossi et al., 2003) in which the basement membrane structure is affected. Thus, ADAMTS18 is required for embryonic lens capsule development from E13.5 onward.

### Adamts18 in lung development

**Fig. 5.**
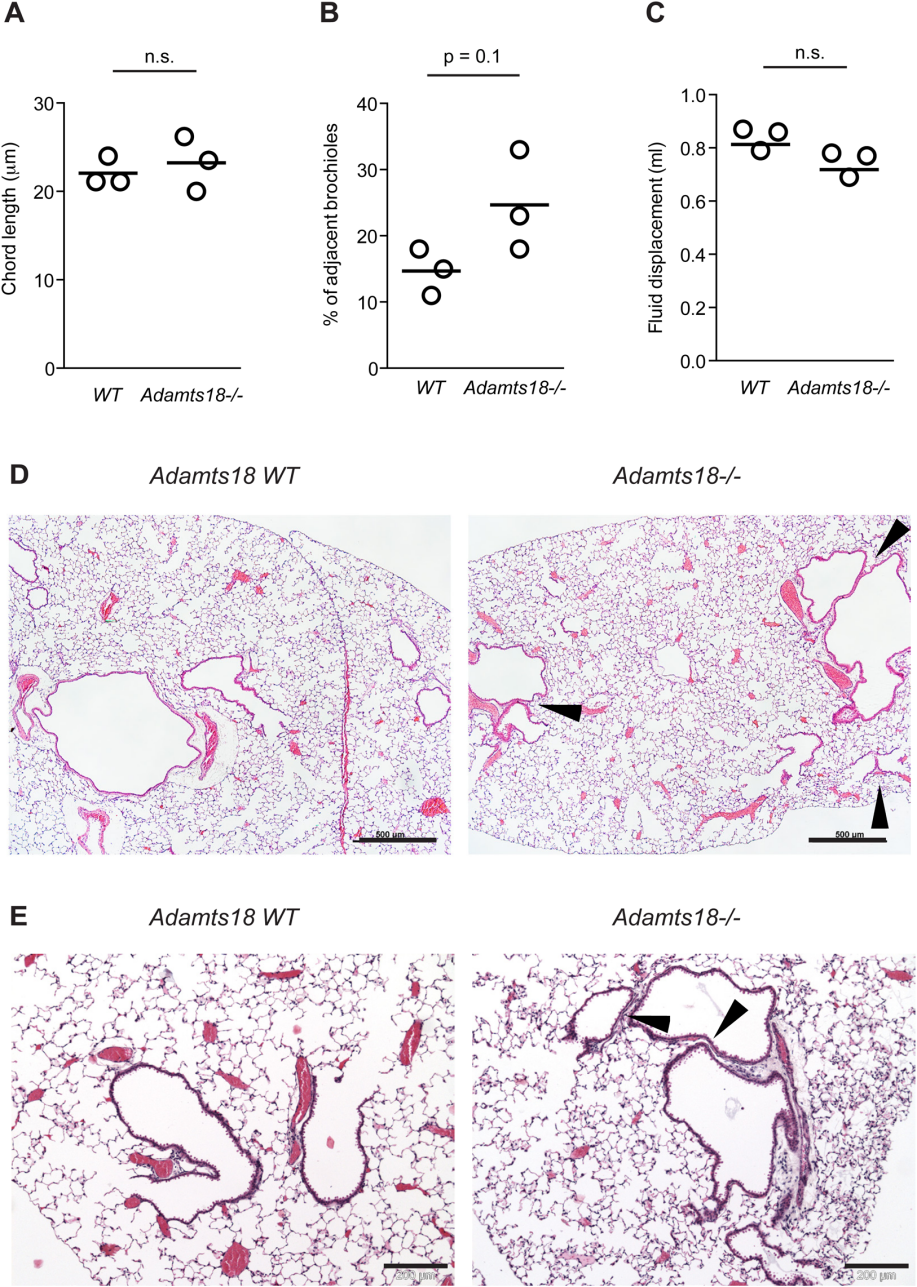
***Adamts18*^−/−^ mice have lung anomalies.** (A) Morphometric analysis of mean linear intercept (chord length) in the lungs of 8-week-old WT and *Adamts18*^−/−^ mice. (B) Morphometric analysis of percentage of adjacent bronchioles in the lungs of 8-week-old WT and *Adamts18*^−/−^ mice. (C) Lung volume of in 8-week-old WT and *Adamts18*^−/−^ mice as measured by fluid displacement in ml. (D,E) Histological sections of lungs from 8-week-old *Adamts18*^−/−^ females and their WT littermates. (D) Low magnification lung micrograph illustrating greater frequency of adjacent airways/bronchioles (arrowheads) in the lungs of *Adamts18^−/−^* mice (−/−) compared to *Adamts18WT* (+/+) littermates. Scale bar: 500 µm. (E) Higher magnification of bronchioles immediately adjacent to each other (arrowheads) in the lungs of *Adamts18*^−/−^ mice. Scale bar: 200 µm.

The lungs reportedly show high expression of *Adamts18* during embryonic development (Cal et al., 2002). To determine whether ADAMTS18 may contribute to lung development and morphology, lungs of 8-week-old females were inflated with fixative at a constant pressure of 20 cm H_2_O, and lung volumes were determined by fluid displacement. Morphological analysis revealed no difference in chord length (Fig. 5A), but a higher percentage of adjacent bronchioles in *Adamts18^−/−^* lungs were observed (Fig. 5B,D,E). Total lung volume tended to be lower in *Adamts18^−/−^* mice, but this finding was not statistically significant (Fig. 5C). Histological analysis of lungs from 11-day-old *Adamts18^−/−^* mice and their WT littermates showed larger airspaces in the mutants with thinner walls between them (Fig. S3A,B). Thus, *Adamts18* has a complex role in lung development and contributes to bronchiolar septation.

### Bleeding time in Adamts18 mutant mice

A thrombin-induced C-terminal 385-amino acid fragment of ADAMTS18 was shown to induce oxidative platelet fragmentation *in vitro*, and an anti-ADAMTS18 antibody injected intravenously shortened the tail vein bleeding time in mice (Li et al., 2009). To assess whether ADAMTS18 was required for coagulation, we measured bleeding time in 6-week-old *Adamts18^−/−^* mice and their WT and heterozygous littermates. We did not observe any statistically significant correlation of the bleeding time with *Adamts18* genotype (Fig. S3C) in 22 mice that were tested.

### Genital anomalies are present in Adamts18^−/−^ females

**Fig. 6.**
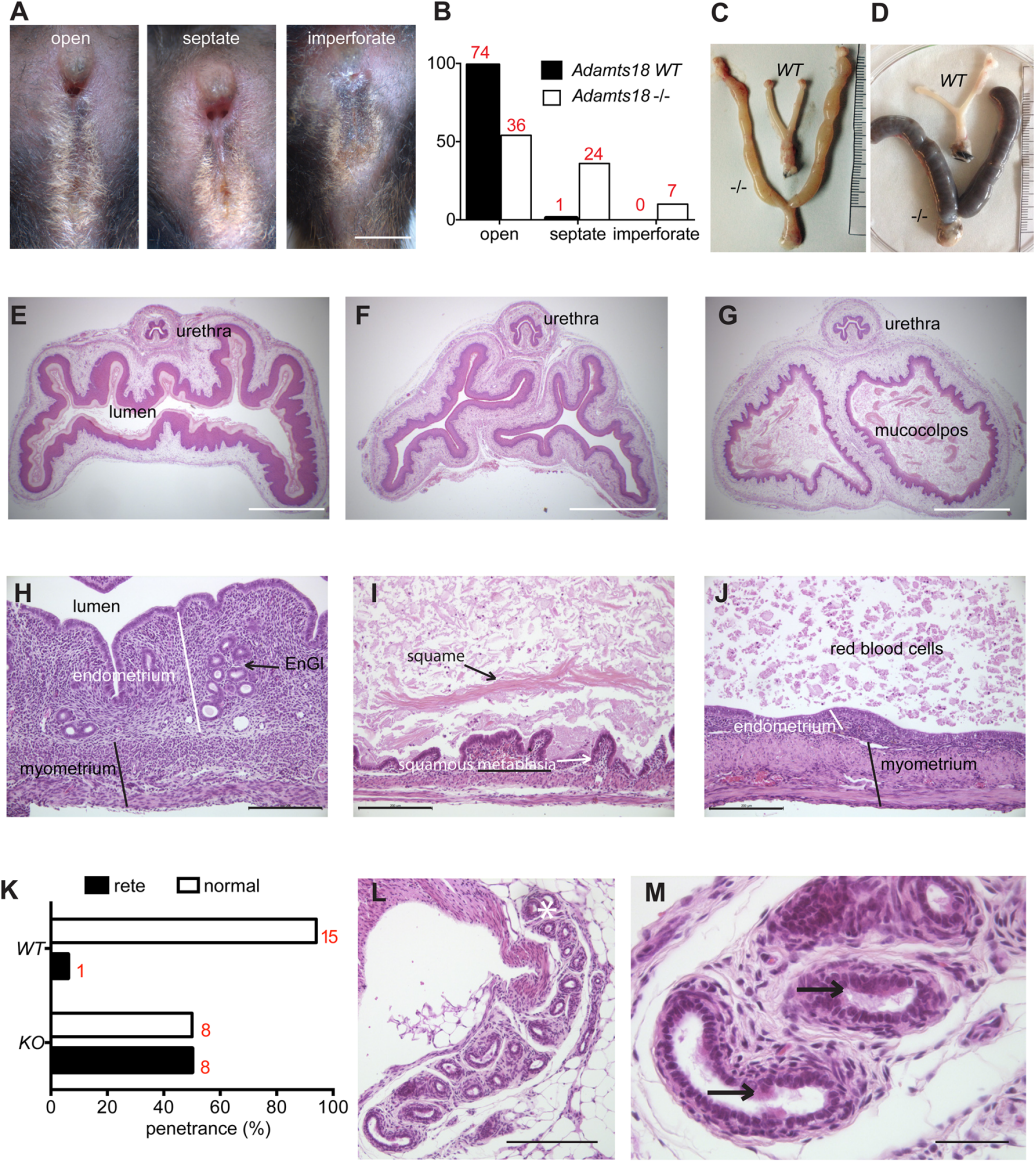
**Female reproductive tract in *Adamts18*^−/−^ females.** (A) Vulvo-vaginal phenotypes observed in *Adamts18*^−/−^ mice. Scale bar: 2 mm. (B) Frequency of vaginal opening defects in 2-month-old *Adamts18*^−/−^ females and their WT littermates (*n*=67 and 76 for *Adamts18*^−/−^ and *WT*, respectively), absolute numbers of females are indicated in red. The incidence of septate and imperforate vaginal phenotypes is significantly different, *P*<0.0001 by Chi-squared test. (C) Mucometra and (D) hemometra in *Adamts18*^−/−^ females with imperforate vagina compared to normal uteri of an age-matched *WT* mice. (E-G) Histologic sections of vaginas of WT (E) and *Adamts18*^−/−^ females (F,G) stained with H&E. Scale bar: 2 mm. (H) Normal uterus histology; H&E-stained. (I) Microscopic appearance of mucometra, with distention and atrophy of the uterine wall and presence of vaginal keratin squames in the lumen (arrow); H&E-stained. (J) Microscopic appearance of hemometra. The uterus is distended as in the previous cases, however mural hemorrhages caused accumulation of red blood cells in the lumen (arrow); H&E-stained. (K) Penetrance of the presence of extraovarian rete in *Adamts18*^−/−^ versus WT mice. (L) Extra ovarian rete within para ovarian fat tissue. Note the mesovarium (white asterisk) adjacent to the convoluted tubules; H&E-stained. (M) Sections of the extraovarian rete lined by cuboidal non-ciliated and columnar ciliated (arrows) epithelium; H&E-stained.

*Adamts18*^−/−^ males bred normally, but 46% of the females were infertile due to imperforate vagina or due to the presence of a variably thick dorsoventral vaginal septum (Fig. 6A). After puberty, the vaginal opening was narrowed by a septum dissecting it dorsoventrally in 36% of the *Adamts18*^−/−^ females, while in another 10% the vagina was still imperforate. Abnormalities of vaginal remodeling have been reported with variable frequency in a several strains and sublines of inbred and outbred mice (Shire, 1984; Sundberg and Brown, 1994; Rodriguez et al., 1997; Ginty and Hoogstraten-Miller, 2008; Chang et al., 2013; Ito et al., 2015). As such, the incidence of the septum was also recorded in WT littermates. Only 1.3% of the WT siblings had a vaginal septum (Fig. 6B). The increased frequency in the *Adamts18*^−/−^ females is hence statistically significant (*P*<0.0001), indicating an effect of the mutant genotype (Fig. 6B).

Vaginal opening is a process that involves apoptosis and occurs at 5 weeks in C57Bl6 mice (Rodriguez et al., 1997). To test whether a difference in apoptosis may account for the phenotype, we performed a terminal deoxynucleotidyl transferase dUTP nick-end labeling (TUNEL) assay on the vagina of four *Adamts18*^−/−^ females and their WT siblings. We failed to detect a significant difference (Fig. S4A,B).

At necropsy, mucocolpos and/or mucometra were observed upon macroscopic inspection in few *Adamts18*^−/−^ females associated with imperforate vagina (Ginty and Hoogstraten-Miller, 2008) (Fig. 6C,D). The dilated uterine horns contained clear to dense fluid, and macrophages, proteinaceous material, exfoliated endometrial cells and occasionally, erythrocytes were detected histologically (Fig. 6J). The presence of thick layers of compact keratin floating within the lumen confirmed that the pathogenesis was related to retention of vaginal fluid and epithelial cells, secondary to a defect in the remodeling of vulvovaginal cavity (Fig. 6I). Rarefaction or even complete loss of endometrial glands, flattening of the luminal uterine epithelium, and thinning of the endometrial and myometrial layers (Fig. 6I,J) are all likely to be secondary to the mechanical pressure exerted by fluid accumulation, leading to dilation and increased mural tension in the uterine horns.

Another morphologic finding with a higher penetrance in *Adamts18*^−/−^ compared to WT females is the presence of a mono- or bilateral extraovarian rete, with 50% vs 6.7% incidence (Fig. 6K). The tubular structure was found within the paraovarian adipose tissue, adjacent to the mesovarium (Fig. 6L). The appearance ranged from a single tubule section to more developed and convoluted forms. The rete was lined by cuboidal non-ciliated and columnar epithelium (Fig. 6M), similar to that described previously (Lee et al., 2011).

## DISCUSSION

We have demonstrated that *Adamts18* has a critical role in the development of a number of organs such as the eye, and specifically, the lens capsule, the lungs, and the female genital tract.

Intriguingly, while *ADAMTS18* mutations are associated with complex eye phenotypes in humans, we discerned a very restricted abnormality in mice, namely fenestration of the lens capsule resulting in posterior extrusions of herniated lens fibers. A similar lens capsule defect and extrusion was reported in mice carrying mutations in perlecan, a basement membrane-specific heparan sulfate proteoglycan core protein, that abrogate the heparin-sulfate attachment sites (Rossi et al., 2003). Lens extrusions with fenestration of the lens capsule have also been seen in *Adamts9* haploinsufficient mice (Dubail and Apte, 2015).

In humans, *ADAMTS18* mutations are associated with pleomorphic ocular manifestations, including microcornea, ectopic pupils, childhood cataract, night blindness, ectopia lentis, rhegmatogenous retinal detachment, and cone-rod dystrophy (Aldahmesh et al., 2013; Peluso et al., 2013; Chandra et al., 2014). In patients carrying *ADAMTS18* mutations that cause eye disease, one reported mutation resulted in premature termination of mRNA at codon 356 and was predicted to result in a null phenotype, whereas the other mutations were more at the C-terminal in the cysteine-rich region of the protease or TSRs (Peluso et al., 2013; Chandra et al., 2014). It will be of interest to explore to what extent abrogation of the enzymatic activity of *Adamts18* accounts for the observed phenotype, and whether the discrepancy between the lens capsule-restricted phenotype of the *Adamts18*^−/−^ eyes and the human *ADAMTS18* mutant eyes relates to domain-specific mutations.

Vaginal opening in mice normally occurs at puberty by means of apoptosis in its caudal-most portion (Rodriguez et al., 1997). The process occurs rapidly, at different ages depending on the mouse strain, and sex hormones play an important role in inducing such remodeling (Nelson et al., 1990; Ito et al., 2015). Impaired remodeling of the vulvovaginal cavity at puberty can lead to an imperforate vagina, or a vaginal septum. The latter is the most common manifestation and has previously been reported in a variety of mouse strains, however with a low frequency (Shire, 1984; Sundberg and Brown, 1994; Rodriguez et al., 1997; Ginty and Hoogstraten-Miller, 2008; Chang et al., 2013; Ito et al., 2015). The observed incidence of vaginal opening defects in *Adamts18^−/−^* is higher than the maximal incidence (11.3%) reported in C57BL/6J WT mice (Gearhart et al., 2004). As such, and given that vaginal septum is a polygenic trait in mice, it is reasonable to consider an effect of the biallelic gene deletion on its pathogenesis. Vaginal septum occurs in humans in a number of different syndromes, some of which have been attributed to specific genetic defects. It can also occur as a sporadic phenotype without an identifiable genetic cause.

ADAMTS proteins have previously been reported to have a role in physiological tissue regression. Combined activity of closely related versican-degrading proteases ADAMTS5, ADAMTS9 and ADAMTS20 is required to enable bone morphogenetic protein (BMP)-induced apoptosis during the regression of the interdigit tissue in mouse distal limbs (McCulloch et al., 2009).

In *Adamts18^−/−^* mice, interdigit tissue regression occurs normally. It is possible that, like versican-degrading ADAMTS proteases, ADAMTS18 alters the cellular microenvironment to create permissive conditions for estrogen-induced apoptosis of vagina cells. We explored this possibility by performing TUNEL assays on the low part of the vagina in a small cohort of *Adamts18^−/−^* mutant and WT mice, but did not detect a difference. It is possible that a larger cohort is needed to discern the phenotype that is not fully penetrant. Alternatively, other mechanisms of cell clearance may be involved in vaginal opening and affected by abrogation of ADAMTS18 function.

Thus, identification of ADAMTS18 substrates is a crucial potential direction in understanding this and other observed phenotypes. Fertility, as assessed by breeding efficiency, another estrogen-dependent characteristic, was normal in *Adamts18^−/−^* females.

*Adamts18^−/−^* females presented an increased incidence of remnants of mesonephric ducts as sections of the extraovarian rete, which could also result from impairment of programmed cell death. The ‘rete’ system consists of three contiguous portions distinguished by their localization. The extraovarian rete is the outermost part of the tubular system and lies within the periovarian soft tissue, then the connecting rete and the intraovarian rete extend into the gonad from the hilus. Rete cells seem to play an important role during gametogenesis, serving as forerunners of the granulosa cells and showing sex hormone-related enzymatic activity (Motta PMM, 1980). The rete system, especially in its extraovarian segment, which is blind-ended and lined by a monolayer of epithelial ciliated and non-ciliated cells, also has a secretory function and as such, it is prone to pathologic changes including cystic dilation in a number of species including humans (Long, 2002; Lee et al., 2011). A secondary negative effect on fertility can occur as consequence of pressure on the ovarian parenchyma from the cyst, leading to disturbance of the cycle and atrophy of the gonad.

In mice, the lungs develop around E9.0 by evagination of the trachea from the anterior foregut endoderm. The main bronchi then appear by expansion of the respiratory epithelium in the surrounding mesoderm by E12.5. During the ensuing pseudoglandular stage (E12.5-E16.5), branching morphogenesis takes place providing a high level of arborization with distinct branching modes (Metzger et al., 2008). ADAMTS18 may contribute to the separation of bronchioles through direct effects on branching mechanisms, or by indirect effects on the subsequent developmental stage, the alveolization, as suggested by the reduced total lung volume trend in the *Adamts18*^−/−^ mice. Systematic examination of the branching pattern from E13 to E16, morphometric analysis at birth, and following alveolization at post-natal day 14, would provide additional insights into the potential mechanisms. At the molecular level, ADAMTS18 may regulate cell-ECM interactions through direct cleavage of cellular adhesion molecules such as integrins and/or components of the ECM, or modification of signaling networks between the developing endoderm and the surrounding mesoderm, or indirectly, by activating or inactivating other proteases or protease inhibitors.

Previous reports implicated ADAMTS18 in platelet function (Li et al., 2009). Our finding that deletion of ADAMTS18 did not affect bleeding time does not preclude such a role. During development, compensatory mechanisms may be activated. For instance, the ADAMTS18 homolog ADAMTS16 may compensate for the absence of ADAMTS18 in mice. As previously noted for closely related ADAMTS proteases, which cooperate in developmental phenotypes, such joint functions with ADAMTS16 will have to be tested by generation of double knockout mice. Alternatively, in the specific genetic background we studied, C57BL/6JOlaHsd, an *Adamts18*-deficient phenotype, may not be fully penetrant. Taken together with genetic findings in humans, our findings indicate that ADAMTS18 should be considered in the context of eye, female reproductive tract abnormalities, and lung anomalies. Presently, little is known about this protease, its substrates and other intermolecular interactions, and such studies may help to elucidate the mechanisms underlying these roles.

## MATERIALS AND METHODS

### Transgenic mice

C57BL/6JOlaHsd inbred mice were purchased from Harlan Laboratories. To generate a conditional targeting vector, an *Aat*II-*Cla*I genomic DNA fragment was excised from BAC BMQ-54M16 (Source Bioscience UK Limited). An h*PGK*-driven neomycin resistance gene flanked by FRT sites and followed by a loxP site was inserted 200 bp before exon 8 and another loxP site was inserted 200 bp after exon 9 (Fig. 1A). The construct was linearized and electroporated into mouse ES cells of 129/SvEv origin (Porret et al., 2006). Correct recombination external to the loxP sites was confirmed by Southern blot analysis. Three correctly recombined clones were selected, injected into C57BL/6N donor blastocysts, and chimeras that transmitted the targeted allele through the germ line were obtained. The mice were crossed to a transgenic mouse carrying an MMTV-CRE transgene, which is expressed in oocytes (Wagner et al., 2001). The progeny of *MMTV-Cre* X *Adamts18^CKO^* with germ line deletion of exons 8 and 9 were backcrossed to inbred C57BL/6JOlaHsd background mice. All mice were maintained and handled according to Swiss guidelines for animal safety, with a 12 h light:12 h dark cycle, controlled temperature, and food and water *ad libitum*. Animal experiments were approved by the ethic veterinary committee of Canton of Vaud, Switzerland (protocols 1541.2 and 1541.3).

### Genotyping and tissue harvesting

Genomic DNA isolated from ear biopsies was used for genotyping by PCR with a combination of three primers for exon8-f: TTCTGGTGCTAACTTGGAACACG, NEO-f: CCAAATGTGTCAGTTTCATAGCCTG, after-loxP-r AGAAGTTAAACCTGGTACCTTCG. The WT allele results in an amplicon of 469 bp and the mutant allele in an amplicon of 227 bp. Mouse tissues were obtained after euthanasia was performed according to Swiss guidelines for animal safety.

### Bleeding time

The tail was cut 2 mm from its tip and touched every 30 s to a sheet of blotting paper. The bleeding time was recorded as the period from time of incision to the time at which no fresh bleeding was identified on the filter paper. Measurements were stopped after 20 min.

### Histopathology and immunohistochemistry

Lungs were prepared for stereological analysis as described previously (Cremona et al., 2013). Briefly, lungs were instilled at a constant pressure of 20 cm H_2_O via an endotracheal cannula with 1.5% paraformaldehyde-1.5% glutaraldehyde in 150 mM HEPES pH 7.35. The trachea was ligated and lungs were placed in fixative. Total lung volume was determined by the water displacement method. Fixed lungs were embedded in 2.5% agar and cut in transverse slices of 1.2 mm. Systematic uniform random sampling was used to pick five lung pieces representative of the whole organ. Following paraffin embedding, 5 µm sections were stained with H&E. About thirty micrographs were captured for each animal using a ColorView IIIu digital camera (Olympus Soft Imaging Solutions) on a Leica DMRB light microscope equipped with an automated motorized stage. The images taken were equally spaced and systematically distributed meander-like over the whole surface of the lung sections. Pictures were quantitatively analyzed by using a test system of lines superimposed over the digital images via the STEPanizer (Tschanz et al., 2011). The Lm was calculated with line counts intersecting septa. All bronchiolar airways were counted and the percentage of adjacent airways was determined. Other tissues were fixed with 4% paraformaldehyde in phosphate-buffered saline (PBS, pH 7.2), embedded in paraffin and cut into 4 µm sections. H&E or PAS stains were performed. The genital system was macro- and microscopically evaluated by a board certified veterinary pathologist.

For immunohistochemistry, anti-β-catenin antibody diluted 1:100 (BD transduction laboratories, Catalog no. 610153, clone 14, lot 15825) was used after antigen retrieval in 10 mM sodium citrate buffer, pH 6.00 at 95°C for 20 min followed by detection with Mouse on Mouse (M.O.M.) immune detection Kit (Vector Laboratories) and mouse IgG Vectastain ABC kit (Vector Laboratories). Sections were counterstained with Mayer's hematoxylin. To detect apoptotic cells, DeadEnd Fluorometric TUNEL System (Promega) was used on 4 µm sections of the lower vagina.

### RNA *in situ* hybridization

Mouse tissue was fixed in 4% PFA overnight at 4°C, processed, and paraffin embedded. Fresh 7 µm sections were used for ISH using RNAscope (all materials from Advanced Cell Diagnostics, Newark, CA) following the manufacturer's protocol. The HybEZ Oven was used for all steps requiring incubation at 40°C. Binding of the specific probe against mouse *Adamts18* mRNA (Catalog no. 452251) was detected with the RNAscope 2.0 HD detection kit ‘RED’. Probes against mouse peptidylprolyl isomerase B (Cyclophilin B, Ppib, Catalog no. 313911) or bacterial dihydrodipicolinate reductase (dapB, Catalog no. 310043) were used as positive and negative controls, respectively. Images were collected using an Olympus BX51 upright microscope, and post-acquisition image processing was performed using Adobe Photoshop. To enhance the red signal in highly pigmented tissues such as the RPE or the iris, the ‘brightness/contrast/intensity’ settings and the ‘hue/saturation’ settings were modulated in order to enhance the red color of the ISH signal and reduce the dark blue colors of the pigment and the hematoxylin counterstain. These adjustments were applied to the entire image.

### RT-PCR

RNA was extracted from mouse tissues with TRIzol reagent (Invitrogen) and further purified with RNA easy kit (Qiagen). *Adamts18* transcript was detected in random hexamer-primed cDNA using SYBR Green FastMix (Quanta) reaction mix and the following primers: Adamts18-r AGCACCGTCCTTTCCAAGTA, Adamts18-f TGTCGTGCCAGTAGAAGTGG. *Adamts18* transcript levels were normalized to the levels of *Rplp0* transcript levels detected with 36b4-f GTGTGTCTGCAGATCGGGTA and 36b4-r CAGATGGATCAGCCAGGAAG. For transcript analysis Adamts18-f CTTAGCCAGTGACAGCGGCAG, Adamts18-r1 GAACATTCTGACCACTTCGACCAG, and Adamts18-r2 CATGTGACCCTGCCTCTAGAATG primers were used.

## References

[BIO019711C1] Aldahmesh M. A., Alshammari M. J., Khan A. O., Mohamed J. Y., Alhabib F. A. and Alkuraya F. S. (2013). The syndrome of Microcornea, Myopic Chorioretinal Atrophy, and Telecanthus (MMCAT) is caused by mutations in ADAMTS18. *Hum. Mutat.* 34, 1195-1199. 10.1002/humu.22374 23818446

[BIO019711C2] Apte S. S. (2009). A Disintegrin-like and Metalloprotease (Reprolysin-type) with Thrombospondin Type 1 Motif (ADAMTS) superfamily: functions and mechanisms. *J. Biol. Chem.* 284, 31493-31497. 10.1074/jbc.R109.052340 19734141 PMC2797218

[BIO019711C3] Cal S., Obaya A. J., Llamazares M., Garabaya C., Quesada V. and López-Otín C. (2002). Cloning, expression analysis, and structural characterization of seven novel human ADAMTSs, a family of metalloproteinases with disintegrin and thrombospondin-1 domains. *Gene* 283, 49-62. 10.1016/S0378-1119(01)00861-7 11867212

[BIO019711C4] Chandra A., Arno G., Williamson K., Sergouniotis P. I., Preising M. N., Charteris D. G., Thompson D. A., Holder G. E., Borman A. D., Davagnanam I. et al. (2014). Expansion of ocular phenotypic features associated with mutations in ADAMTS18. *JAMA Ophthalmol.* 132, 996-1001. 10.1001/jamaophthalmol.2014.940 24874986

[BIO019711C5] Chang T. K., Ho P., Liang C. T. and Yu C. K. (2013). Effects of vaginal septa on the reproductive performance of BALB/cByJNarl mice. *J. Am. Assoc. Lab. Anim. Sci.* 52, 520-523. 24041204 PMC3784654

[BIO019711C7] Colige A., Nuytinck L., Hausser I., van Essen A. J., Thiry M., Herens C., Adès L. C., Malfait F., Paepe A. D., Franck P. et al. (2004). Novel types of mutation responsible for the dermatosparactic type of Ehlers–Danlos syndrome (Type VIIC) and common polymorphisms in the ADAMTS2 gene. *J. Invest. Dermatol.* 123, 656-663. 10.1111/j.0022-202X.2004.23406.x 15373769

[BIO019711C42] Cremona T. P., Tschanz S. A., von Garnier C., Benarafa C. (2013). SerpinB1 deficiency is not associated with increased susceptibility to pulmonary emphysema in mice. *Am J. Physiolo Lung Cell Mol. Physiol.* 305, L981-L989. 10.1152/ajplung.00181.2013 24163143

[BIO019711C8] Dagoneau N., Benoist-Lasselin C., Huber C., Faivre L., Mégarbané A., Alswaid A., Dollfus H., Alembik Y., Munnich A., Legeai-Mallet L. et al. (2004). ADAMTS10 mutations in autosomal recessive Weill-Marchesani syndrome. *Am. J. Hum. Genet.* 75, 801-806. 10.1086/425231 15368195 PMC1182109

[BIO019711C9] Danysh B. P. and Duncan M. K. (2009). The lens capsule. *Exp. Eye Res.* 88, 151-164. 10.1016/j.exer.2008.08.002 18773892 PMC2674021

[BIO019711C10] Donley N. and Thayer M. J. (2013). DNA replication timing, genome stability and cancer: late and/or delayed DNA replication timing is associated with increased genomic instability. *Semin. Cancer Biol.* 23, 80-89. 10.1016/j.semcancer.2013.01.001 23327985 PMC3615080

[BIO019711C11] Dubail J. and Apte S. S. (2015). Insights on ADAMTS proteases and ADAMTS-like proteins from mammalian genetics. *Matrix Biol.* 44-46, 24-37. 10.1016/j.matbio.2015.03.001 25770910

[BIO019711C12] Enomoto H., Nelson C. M., Somerville R. P. T., Mielke K., Dixon L. J., Powell K. and Apte S. S. (2010). Cooperation of two ADAMTS metalloproteases in closure of the mouse palate identifies a requirement for versican proteolysis in regulating palatal mesenchyme proliferation. *Development* 137, 4029-4038. 10.1242/dev.050591 21041365 PMC2976286

[BIO019711C13] Gearhart S., Kalishman J., Melikyan H., Mason C. and Kohn D. F. (2004). Increased incidence of vaginal septum in C57BL/6J mice since 1976. *Comp. Med.* 54, 418-421. 15357323

[BIO019711C14] Ginty I. and Hoogstraten-Miller S. (2008). Perineal swelling in a mouse. Diagnosis: imperforate vagina with secondary mucometra. *Lab. Anim.* 37, 196-199. 10.1038/laban0508-196 PMC2597298 18431391

[BIO019711C15] Ito T., Bai T., Tanaka T., Yoshida K., Ueyama T., Miyajima M., Negishi T., Kawasaki T., Takamatsu H., Kikutani H. et al. (2015). Semaphorin 4D induces vaginal epithelial cell apoptosis to control mouse postnatal vaginal tissue remodeling. *Mol. Med. Rep.* 11, 829-836. 10.3892/mmr.2014.2773 25351707 PMC4262505

[BIO019711C16] Kallifatidis G., Boros J., Shin E. H. H., McAvoy J. W. and Lovicu F. J. (2011). The fate of dividing cells during lens morphogenesis, differentiation and growth. *Exp. Eye Res.* 92, 502-511. 10.1016/j.exer.2011.03.012 21440542 PMC3137915

[BIO019711C17] Kelwick R., Desanlis I., Wheeler G. N. and Edwards D. R. (2015). The ADAMTS (A Disintegrin and Metalloproteinase with Thrombospondin motifs) family. *Genome Biol.* 16, 113 10.1186/s13059-015-0676-3 26025392 PMC4448532

[BIO019711C18] Kuno K., Kanada N., Nakashima E., Fujiki F., Ichimura F. and Matsushima K. (1997). Molecular cloning of a gene encoding a new type of metalloproteinase-disintegrin family protein with thrombospondin motifs as an inflammation associated gene. *J. Biol. Chem.* 272, 556-562. 10.1074/jbc.272.1.556 8995297

[BIO019711C19] Lee S.-H., Ichii O., Otsuka S., Elewa Y. H., Namiki Y., Hashimoto Y. and Kon Y. (2011). Ovarian cysts in MRL/MpJ mice are derived from the extraovarian rete: a developmental study. *J. Anat.* 219, 743-755. 10.1111/j.1469-7580.2011.01431.x 21951275 PMC3237882

[BIO019711C20] Levy G. G., Nichols W. C., Lian E. C., Foroud T., McClintick J. N., McGee B. M., Yang A. Y., Siemieniak D. R., Stark K. R., Gruppo R. et al. (2001). Mutations in a member of the ADAMTS gene family cause thrombotic thrombocytopenic purpura. *Nature* 413, 488-494. 10.1038/35097008 11586351

[BIO019711C21] Li Z., Nardi M. A., Li Y.-S., Zhang W., Pan R., Dang S., Yee H., Quartermain D., Jonas S. and Karpatkin S. (2009). C-terminal ADAMTS-18 fragment induces oxidative platelet fragmentation, dissolves platelet aggregates, and protects against carotid artery occlusion and cerebral stroke. *Blood* 113, 6051-6060. 10.1182/blood-2008-07-170571 19218546 PMC2699219

[BIO019711C22] Long G. G. (2002). Apparent mesonephric duct (rete anlage) origin for cysts and proliferative epithelial lesions in the mouse ovary. *Toxicol. Pathol.* 30, 592-598. 10.1080/01926230290105785 12371668

[BIO019711C23] McCulloch D. R., Nelson C. M., Dixon L. J., Silver D. L., Wylie J. D., Lindner V., Sasaki T., Cooley M. A., Argraves W. S. and Apte S. S. (2009). ADAMTS metalloproteases generate active versican fragments that regulate interdigital web regression. *Dev. Cell* 17, 687-698. 10.1016/j.devcel.2009.09.008 19922873 PMC2780442

[BIO019711C24] Metzger R. J., Klein O. D., Martin G. R. and Krasnow M. A. (2008). The branching programme of mouse lung development. *Nature* 453, 745-750. 10.1038/nature07005 18463632 PMC2892995

[BIO019711C25] Morales J., Al-Sharif L., Khalil D. S., Shinwari J. M. A., Bavi P., Al-Mahrouqi R. A., Al-Rajhi A., Alkuraya F. S., Meyer B. F. and Al Tassan N. (2009). Homozygous mutations in ADAMTS10 and ADAMTS17 cause lenticular myopia, ectopia lentis, glaucoma, spherophakia, and short stature. *Am. J. Hum. Genet.* 85, 558-568. 10.1016/j.ajhg.2009.09.011 19836009 PMC2775842

[BIO019711C26] Motta PMM H. E. (1980). *The Biology of the Ovary*, Vol. 2, p 9 The Hague: Martinus Nijhoff Publishers.

[BIO019711C27] Nelson J. F., Karelus K., Felicio L. S. and Johnson T. E. (1990). Genetic influences on the timing of puberty in mice. *Biol. Reprod.* 42, 649-655. 10.1095/biolreprod42.4.649 2346773

[BIO019711C28] Peluso I., Conte I., Testa F., Dharmalingam G., Pizzo M., Collin R. W. J., Meola N., Barbato S., Mutarelli M., Ziviello C. et al. (2013). The ADAMTS18 gene is responsible for autosomal recessive early onset severe retinal dystrophy. *Orphanet J. Rare Dis.* 8, 16 10.1186/1750-1172-8-16 23356391 PMC3568033

[BIO019711C29] Porret A., Merillat A. M., Guichard S., Beermann F. and Hummler E. (2006). Tissue-specific transgenic and knockout mice. *Methods Mol. Biol.* 337, 185-205. 16929948 10.1385/1-59745-095-2:185

[BIO019711C30] Richard S., Winston K., Simon J. (2001). Ocular Development. In: *Systematic Evaluation of the Mouse Eye* (ed. Smith R. S., Simon W. M., Patsy J., Nishina M., Sundberg J. P.), pp. 45-66. Boca Raton, Florida, USA: CRC Press.

[BIO019711C31] Rodriguez I., Araki K., Khatib K., Martinou J.-C. and Vassalli P. (1997). Mouse vaginal opening is an apoptosis-dependent process which can be prevented by the overexpression of Bcl2. *Dev. Biol.* 184, 115-121. 10.1006/dbio.1997.8522 9142988

[BIO019711C32] Rossi M., Morita H., Sormunen R., Airenne S., Kreivi M., Wang L., Fukai N., Olsen B. R., Tryggvason K. and Soininen R. (2003). Heparan sulfate chains of perlecan are indispensable in the lens capsule but not in the kidney. *EMBO J.* 22, 236-245. 10.1093/emboj/cdg019 12514129 PMC140094

[BIO019711C33] Seidah N. G., Mayer G., Zaid A., Rousselet E., Nassoury N., Poirier S., Essalmani R. and Prat A. (2008). The activation and physiological functions of the proprotein convertases. *Int. J. Biochem. Cell Biol.* 40, 1111-1125. 10.1016/j.biocel.2008.01.030 18343183

[BIO019711C34] Shieh H.-S., Mathis K. J., Williams J. M., Hills R. L., Wiese J. F., Benson T. E., Kiefer J. R., Marino M. H., Carroll J. N., Leone J. W. et al. (2008). High resolution crystal structure of the catalytic domain of ADAMTS-5 (aggrecanase-2). *J. Biol. Chem.* 283, 1501-1507. 10.1074/jbc.M705879200 17991750

[BIO019711C35] Shire J. G. M. (1984). Studies on the inheritance of vaginal septa in mice, a trait with low penetrance. *Reproduction* 70, 333-339. 10.1530/jrf.0.0700333 6694148

[BIO019711C36] Stanton H., Melrose J., Little C. B. and Fosang A. J. (2011). Proteoglycan degradation by the ADAMTS family of proteinases. *Biochim. Biophys. Acta* 1812, 1616-1629. 10.1016/j.bbadis.2011.08.009 21914474

[BIO019711C37] Sundberg J. P. and Brown K. S. (1994). Imperforate vagina and mucometra in inbred laboratory mice. *Lab. Anim. Sci.* 44, 380-382. 7983855

[BIO019711C38] Tschanz S. A., Burri P. H. and Weibel E. R. (2011). A simple tool for stereological assessment of digital images: the STEPanizer. *J. Microsc.* 243, 47-59. 10.1111/j.1365-2818.2010.03481.x 21375529

[BIO019711C39] Wagner K.-U., Ward T., Davis B., Wiseman R. and Hennighausen L. (2001). Spatial and temporal expression of the Cre gene under the control of the MMTV-LTR in different lines of transgenic mice. *Transgenic Res.* 10, 545-553. 10.1023/A:1013063514007 11817542

[BIO019711C40] Xiong D.-H., Liu X.-G., Guo Y.-F., Tan L.-J., Wang L., Sha B.-Y., Tang Z.-H., Pan F., Yang T.-L., Chen X.-D. et al. (2009). Genome-wide association and follow-up replication studies identified ADAMTS18 and TGFBR3 as bone mass candidate genes in different ethnic groups. *Am. J. Hum. Genet.* 84, 388-398. 10.1016/j.ajhg.2009.01.025 19249006 PMC2667986

[BIO019711C41] Zander C. B., Cao W. and Zheng X. L. (2015). ADAMTS13 and von Willebrand factor interactions. *Curr. Opin. Hematol.* 22, 452-459. 10.1097/MOH.0000000000000169 26186678 PMC4824554

